# An obesity paradox in preterm birth: A case control study

**DOI:** 10.1371/journal.pone.0321876

**Published:** 2025-05-08

**Authors:** Krystal Hunter, Michael Ehrlich, Jocelyn Mitchell-Williams

**Affiliations:** 1 Department of Medicine, Cooper Medical School of Rowan University, Camden, New Jersey, United States of America; 2 Cooper Hospital Research Institute, Cooper Hospital, Camden, New Jersey, United States of America; 3 Martin Tuchman School of Management, New Jersey Institute of Technology, Newark, New Jersey, United States of America; 4 Obstetrics and Gynecology, Cooper Medical School of Rowan University, Camden, New Jersey, United States of America; 5 Cooper Hospital Department of Obstetrics and Gynecology, Cooper Hospital, Camden, New Jersey, United States of America; University of the Witwatersrand, SOUTH AFRICA

## Abstract

The March of Dimes Global action report indicated that preterm birth (PTB) rates are increasing in most countries. It is the most important cause of neonatal deaths and the second leading cause of death in children under age 5. Literature reporting the relationship between maternal pre-pregnancy body mass index (BMI) and PTB has previously yielded inconsistent conclusions. Our objective is to fill in the knowledge gap by evaluating the interaction of socio-economic status (SES) and BMI and its relationship to the rate of PTB. This is a case control study using the Natality Data of the National Vital Statistics System from the years 2020–2022. BMI was a significant factor in PTB for lower socioeconomic status (LSES) women. For every increase in BMI, there was a decrease in the probability of PTB (OR = 0.923, 95% CI 0.915–0.931, P < 0.001). Those who were LSES also had a curved relationship with PTB indicating that the as BMI increases, the odds of PTB decreases up until a BMI value, then the PTB rate increases. This relationship was not found in higher economic status women. Our study had two significant findings. We first found an obesity paradox in PTB for those mothers who are LSES. We also found that the relationship between BMI and PTB was not linear but curvilinear, bridging the gap in the conclusions of other studies. This study fills in the knowledge gap of BMI and PTB by adding the consideration of social class and by creating a polynomial BMI term.

## Introduction

According to the March of Dimes Global action report, preterm birth (PTB) rates are on the rise in most countries. In 2014, it was estimated that of 38 nations with high-quality data, 26 (68.4%) had increased rates of preterm birth from 2000 to 2014 [1]. In 2018, the World Health Organization reported that of 65 countries with reliable trend data, all but three showed an increase in preterm birth rates over the past 20 years [2]. It is now the single most important cause of neonatal deaths (babies under 28 days) and the second leading cause of death in children under age five. In 2018, the incidence of PTB in the United States increased for the fourth year in a row to 10.02% [3] with an estimated annual cost of $26.2B [4].

The effect of maternal body mass index (BMI) on the rate of preterm birth is inconclusive in literature. Several studies show that the rate of PTB increases as BMI increases [5–7]. It has been found that obese women are more likely to have infants with congenital anomalies and that these infants are more likely to have been delivered preterm. Women with higher BMI have been found to have a higher risk of pre-eclampsia, diabetes, and indicated preterm births [8]. Khatibi et al reported that maternal BMI greater than normal weight (BMI 18.5–24.9) was associated with an increased odds of PTB with adjusted odds ratio (aOR) ranging from 1.11 (95% confidence interval [CI], 1.03–1.20) for pre-obesity/overweight (BMI 25–29.9) to 2.00 (95% CI, 1.48–2.71) for grade-III obesity (BMI greater than 40) [9]. With the use of seven complementary methods of two-sample Mendelian randomization analysis, Kurtz et al found evidence for the causal association between higher BMI and preterm birth: elevated BMI was associated with greater risk of preterm birth (odds ratio [OR] per SD = 1.50; 95% CI 1.11–2.03) [10]. There is also literature indicating high BMI is protective against PTB [11–13]. Hendler et al reported decreased rates of PTB as BMI increases. Those who were underweight (BMI less than 19) had 16.6% spontaneous PTB, normal weight (BMI 19–24.9) had 11.3%, overweight (BMI 25–29.9) having 8.1% and obese (BMI greater than 30) a composited 6.2 rate [14]. The association between being underweight and PTB can possibly be a result of maternal thinness being associated with reduced uterine blood flow resulting from insufficient consumption of necessary nutrients [5].

The study of socioeconomic status (SES) and PTB is diverse because of the many elements that comprise SES. There were studies that used the type of neighborhood to measure SES. Some of these studies found that neighborhood was a significant factor in PTB, with those living in more disadvantaged neighborhoods having higher odds [15,16]. In the meta-analysis by Ncube et al, it was found that there was 27% greater risk of PTB in disadvantaged neighborhoods when compared with less disadvantaged geographical areas [17]. In contrast, Clayborne et al did not show a significant relationship between neighborhood SES and birth outcomes including PTB [18].

There were other studies that used income as a measure of SES [19,20]. Pitts et al found that PTB rates per 1,000 were 104.6 for the affluent group, 108.9 for the upper-middle group, 115.8 for the lower-middle group, and 146.9 per 1,000 for the poverty group yielding a significant difference between the income classes [21]. Years of education was used to measure SES in other studies that found that those who had lower education had greater odds of PTB [22,23]. In the study by Morgen et al, education was a stronger factor in PTB than income and employment status. Those who were less educated (less than 10 years of schooling) had a 22% greater hazard of PTB for those nulliparous and 56% greater hazard for those who were parous [24]. In another study by Mohlman et al, it was found that women with a high school education or less represented more than 50% of the PTB’s [25].

While the intersection of weight and SES has not been addressed in the context of PTB, there is exhaustive literature on the comparison between these two in a general context. Paeratakul et al found that there was a relationship between SES and perception of being overweight (PSOW). Those who had higher income had a significantly higher PSOW than those of a lower income. Those with higher education had 1.6 greater odds of PSOW than those of lower education [26]. Sobal et al reported that in developing countries, there was a strong inverse relationship among women. Obesity was six times more prevalent among women of LSES than among those of HSES, while there was a direct relationship observed in developing countries [27].

With rates of PTB increasing in the United States and the world, it is important to examine factors that may contribute to its increased incidence. There are two aims of this paper. The first objective was to contribute to the literature by evaluating the relationship between BMI and PTB, as well as the relationship between BMI, PTB, and SES. This would add to the knowledge because many studies report BMI and SES as factors in PTB, but none explores the interaction of these two factors. Also, this would provide evidence that social determinants of health

do not work in isolation but connect to each other. By examining the effect of BMI and SES on PTB, we are examining two important factors that will enable widening knowledge. The second objective is to show that BMI has a curvilinear relationship demonstrating an optimal weight where the odds of PTB are at their lowest. This would contribute to literature because many studies assume a general linear relationship between BMI and PTB. Demonstration of a curvilinear relationship may fill in the gap between studies with inconsistent relationships between BMI and PTB.

## Methods

Data for this study was received from the Natality Data within the National Vital Statistics System of the National Center for Health Statistics. This system provides demographic and health data for births occurring during the calendar year. The microdata is based on information abstracted from birth certificates filed in vital statistics offices of each state and District of Columbia -. This study was reviewed by the Cooper University Healthcare Institutional Review Board and met criteria for exempt status. There were 10,965,783 records within the files for all years. Included in the study were records of those mothers between the ages of 25–45 who had singleton births. We chose the lower limit age of 25 because we used education to determine SES. We wanted to be sure that we included those who could possibly complete at least a bachelor’s degree. If those younger were included, there is risk of counting them as less educated when they were too young to reasonably complete higher education. We excluded those who were over 45 because according to a July 2018 FAQ document (FAQ060), by the age of 45, fertility declines to the point that getting pregnant naturally is unlikely for most women. We also excluded those who had undefined or unknown insurance, BMI, education, race, prenatal care, marital status, gravity, parity, hypertension (HTN) and diabetes (DM).

We then created a separate file that only had PTB records to represent our cases. We then randomly created a file that had the number of controls that matched the number of records in the PTB file. When all was consolidated there were a total of 1,255, 866 records.

The primary data points that we examined were PTB, BMI and SES. We looked at BMI as a continuous variable. There was not a PTB variable within the file, so we used gestational age at birth to create the “PTB” data element. We coded PTB as a “yes” if the gestational age at birth was less than 37 weeks and “no” if greater than or equal to 37 weeks. HSES was coded as “yes” if the subject had both a bachelor’s degree or greater and private insurance. LSES was coded as “yes” if the subject had a high school diploma or less and were recipients of WIC and Medicaid insurance. Our controlling variables included age, weight gain, marital status, previous births, prenatal care, hypertension, diabetes and race.

### Statistical methods

We used three logistic regression models: One for full data, HSES women only and LSES women only. The dependent variable was PTB. The controlling variables served as the independent variables. BMI, age and weight gain were given square terms in order to examine whether there were curvilinear relationships with PTB. We used SAS 9.4 (Cary, NC) and SPSS 27 (IBM, Armonk, NY) for analysis. This study was exempt from institutional review board (IRB) review because the data used was publicly available.

## Results

**Fig 1:**
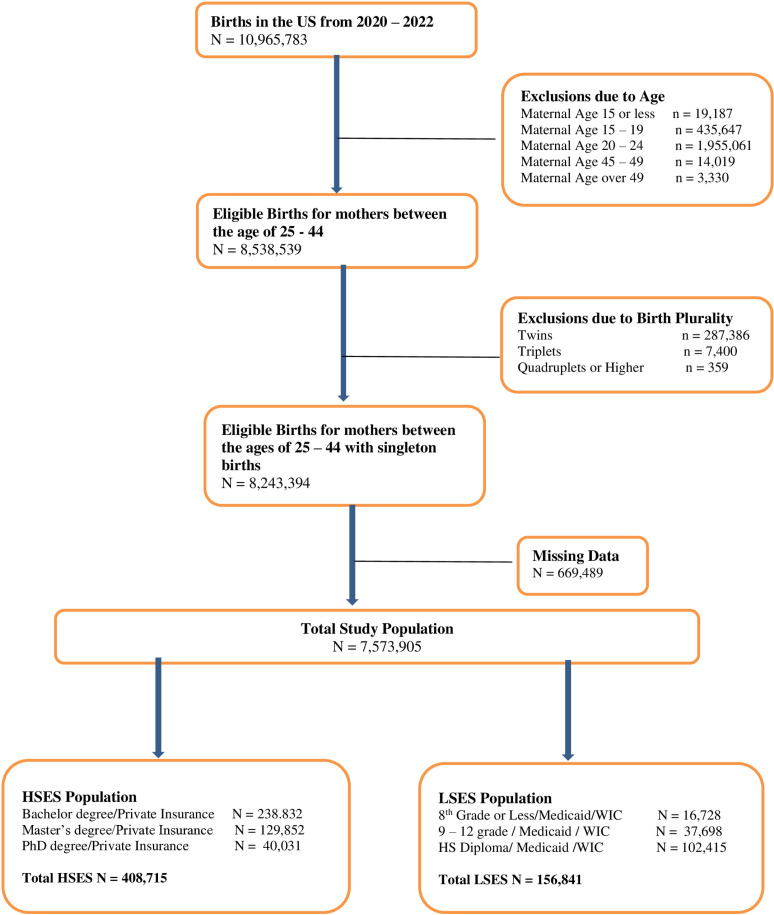
Diagram of study population.

**Fig 2:**
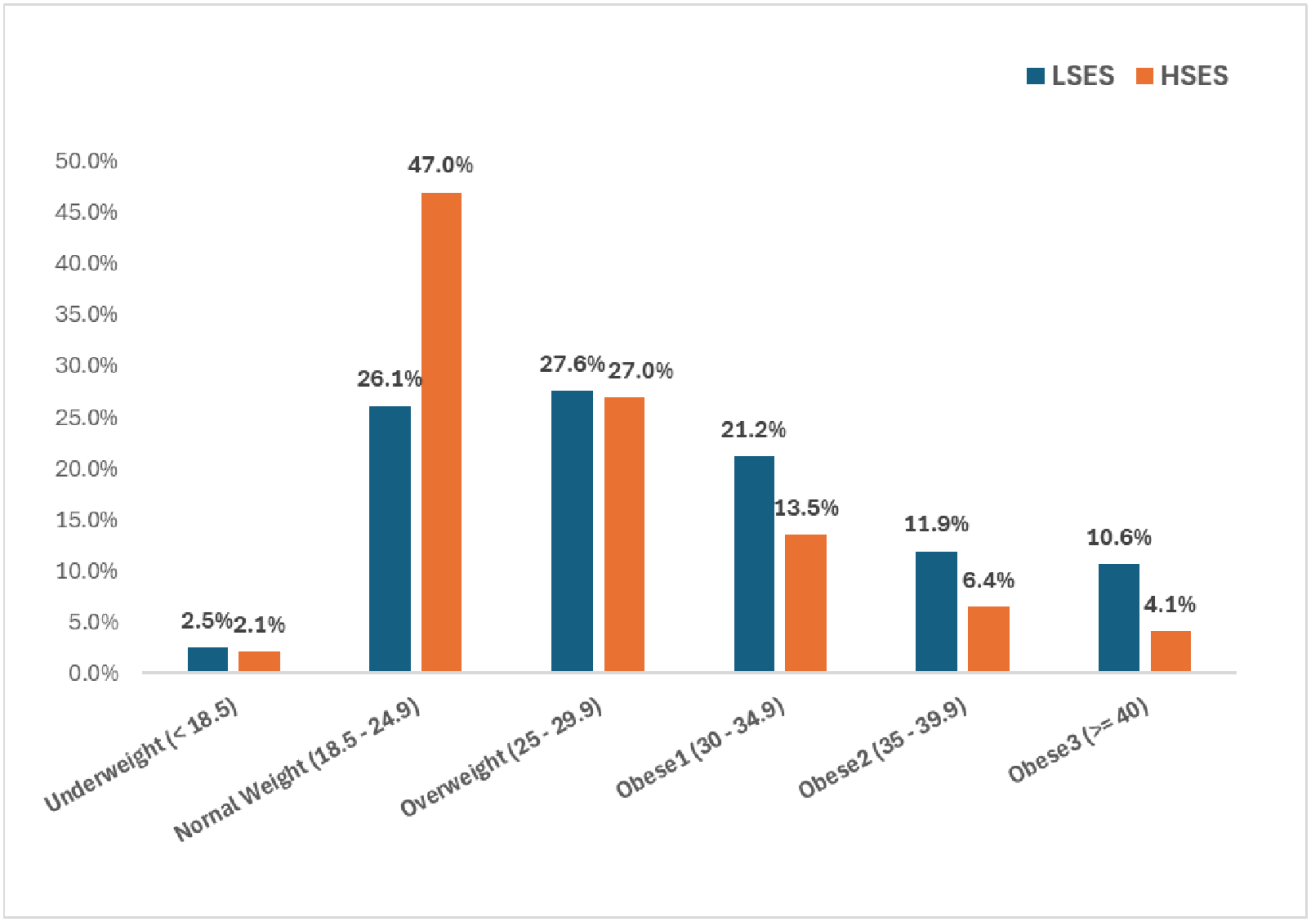
BMI groups by SES.

There were 1,255,866 records used for analysis. After accounting for education level and insurance status, there were 408,715 coded as HSES and 156,841 coded as LSES (Fig 1). Baseline characteristics are seen in Tables 1 and 2. The average age in our overall sample was 31.76 + /- 4.42. The average age was 32.86 + /- 4.08 for those who were HSES and 31 + /- 4.57 for those who were LSES. The average BMI in our overall sample, HSES and LSES were 28.38 + /- 7.21, 26.59 + /- 6.23 and 29.93 + /- 7.76 respectively. The rate of PTB was 43.4% for HSES sample and 55.9% of the LSES sample. The rates of weight groups between HSES and LSES samples were as follows: normal weight – 47% vs 26.2% respectively; overweight – 27% vs 27.6% respectively; obese (all grades) 24% vs 43.7% respectively. (see Fig 2 for the BMI Groups By SES) In our overall sample, 51.1% were White, 15.6% were Black, 23.2% were Hispanic and 10.1% were of other races.

**Table 1:** Baseline characteristics of overall population.

	N	
Maternal Age (Mean/SD)	1,255,866	31.76 ± 4.42
Maternal BMI (Mean/SD)	1,255,866	28.38 ± 7.21
Maternal Education (n/ %)	1,255,866	
*HS Diploma or Less*		396,904 (31.6%)
*Some College*		237,747 (18.9%)
*Associate’s Degree*		122,173 (9.7%)
*Bachelor’s Degree*		305,803 (24.3%)
*Master’s Degree*		149,206 (11.9%)
*PhD Degree*		44,033 (3.5%)
Insurance (n/ %)	1,255,866	
*Medicaid*		473,261 (37.7%)
*Private Insurance*		697,359 (55.5%)
*Self-Pay*		44,727 (3.6%)
*Other*		40,519 (3.2%)
Pregnancy Weight Gain (Mean/SD)	1,255,866	27.13 ± 15.13
Married (n/ %)	1,116,776	730,677 (65.4%)
Previous Births (n/ %)	1,255,866	937,060 (74.6%)
Prenatal Care Initiation (n/ %)	1,255,866	
*First Trimester*		999,795 (79.6%)
*Second Trimester*		179,034 (14.3%)
*Third Trimester*		43,221 (3.4%)
*No Prenatal Care*		33,816 (2.7%)
Pre-existing HTN (n/ %)	1,255,866	63, 724 (5.1%)
Gestational HTN (n/ %)	1,255,866	171,561 (13.7%)
Pre-existing DM (n/ %)	1,255,866	30,119 (2.4%)
Gestational DM (n/ %)	1,255,866	133, 527 (10.6%)
Race (n/ %)	1,255,866	
*White*		641,623 (51.1%)
*Black*		196,230 (15.6%)
*Hispanic*		291,745 (23.2%)
*Other Races*		126,268 (10.1%)

**Table 2:** Baseline characteristics of HSES/LSES population.

	HSES		LSES	
N		N	
Maternal Age (Mean/SD)	408,715	32.86 ± 4.08	156,841	31 ± 4.57
Maternal BMI (Mean/SD)	408,715	26.59 ± 6.23	156,841	29.93 ± 7.76
Maternal Education (n/ %)^*^	408,715		156,841	
*HS Diploma or Less*				156,841 (100%)
*Some College*				
*Associate’s Degree*				
*Bachelor’s Degree*		238,832 (58.4%)		
*Master’s Degree*		129,852 (31.8%)		
*PhD Degree*		40,031 (9.8%)		
Insurance (n/ %)^*^	408,715		156,841	
*Medicaid*				156,841 (100%)
*Private Insurance*		408,715 (100%)		
Pregnancy Weight Gain (Mean/SD)	408,715	28.90 ± 13.45	156,841	24.81 ± 16.07
Married (n/ %)	362,546	331,753 (91.5%)	135,604	47,866 (35.3%)
Previous Births (n/ %)	408,715	251,151 (61.4%)	156,841	139,167 (88.7%)
Prenatal Care Initiation (n/ %)	408,715		156,641	
*First Trimester*		368,074 (90.1%)		109,629 (69.9%)
*Second Trimester*		31,394 (7.7%)		34,250 (21.8%)
*Third Trimester*		6,255 (1.5%)		7,991 (5.1%)
*No Prenatal Care*		2,992 (0.7%)		4,971 (3.2%)
Pre-existing HTN (n/ %)	408,715	5,547 (3.9%)	156,641	6,140 (3.9%)
Gestational HTN (n/ %)	408,715	38,280 (9.4%)	156,641	19,670 (12.5%)
Pre-existing DM (n/ %)	408,715	15,112 (3.7%)	156,641	10,112 (6.4%)
Gestational DM (n/ %)	408,715	54,026 (13.2%)	156,641	20,379 (13%)
Race (n/ %)	408,715		156,641	
*White*		38,280 (68.8%)		42,573 (27.1%)
*Black*		15,112 (6.9%)		36,856 (23.5%)
*Hispanic*		39,860 (9.8%)		67,604 (43.1%)
*Other Races*		59,311 (14.5%)		9,808 (6.3%)
Preterm Births (n/%)	408,715	177,265 (43.4%)	156,841	87,622 (55.9%)

* The dark boxes are placed in areas where the data reported is not applicable. For those of higher socioeconomic status (HSES) only college and above applies for education and private and self-pay only applies for insurance. For those of lower socioeconomic status, only HS diploma or less applies for education and Medicaid and self –pay apply for insurance.

In Table 3, our regression model for the overall sample showed that there was a significant relationship between BMI and PTB after considering all secondary factors. For every unit increase in BMI, there were decreased odds of PTB (OR = 0.965, 95% CI 0.961–0.968, P < 0.001). The square term of BMI was also significant indicating a curvilinear relationship with PTB.

**Table 3:** Logistic regression for overall population.

Predictor	β	P-Value	Odds Ratio	95% Confidence Interval
Constant	3.262	<0.001		
Age	−0.083	<0.001	0.920	0.909–0.931
Age Squared	0.002	<0.001	1.002	1.001–1.002
BMI	−0.036	<0.001	0.965	0.961–0.968
BMI Squared	<0.000	<0.001	1.000	1.000–1.001
Weight (Wt) Gain	−0.039	<0.001	0.962	0.961–0.963
Weight Gain	<0.001	<0.001	1.000	1.000–1.000
Married	−0.302	<0.001	0.739	0.733–0.746
Previous Births	−0.068	<0.001	0.935	0.926–0.943
Prenatal Care Initiation (Reference = No Prenatal Care)				
*First Trimester*	−1.040	<0.001	0.353	0.344–0.363
*Second Trimester*	−1.136	<0.001	0.321	0.312–0.330
*Third Trimester*	−1.324	<0.001	0.266	0.257–0.275
HTN	1.133	<0.001	3.105	3.071–3.139
DM	0.361	<0.001	1.435	1.417–1.452
Race (Reference = White)				
*Black*	0.355	<0.001	1.426	1.409–1.443
*Hispanic*	0.133	<0.001	1.142	1.130–1.154
*Other Races*	0.069	<0.001	1.072	1.057–1.087

**Fig 3:**
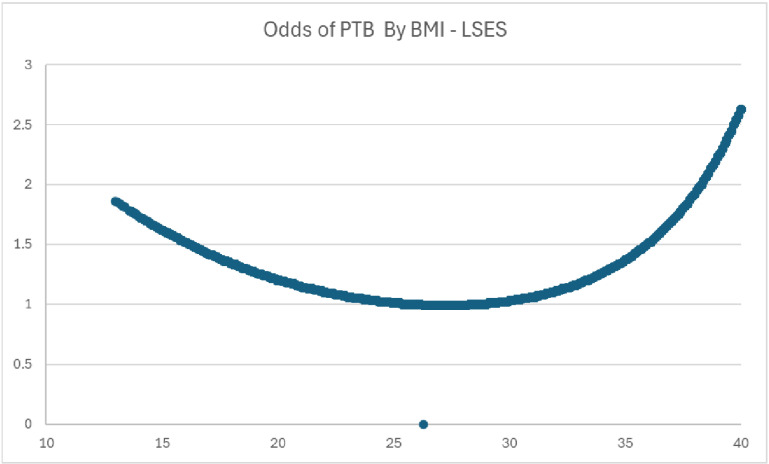
BMI curve for LSES. Curve derived from logit formula: logb p/1 – p = β0 + βBMIX + βBMISqX2. (β0 = constant + all variables*mean or percentage values). Odds were derived taking the exponent of the logit result. Probability = 1/1 + odds.

The regression models for the two SES’s are seen in Tables 4 and 5. For those who are HSES, BMI was not significant. The dynamic was very different for those who were LSES, where BMI was a significant factor in PTB. For every increase in BMI, there was a decrease in the probability of PTB (OR = 0.923, 95% CI 0.915–0.931, P < 0.001). As with the overall sample, those who were LSES also had a curved relationship with PTB. See Fig 3 for the BMI Curve for LSES which shows that as BMI increases the odds of PTB decreases until the BMI has a value of approximately 26–32.5 where the probability of PTB starts to increase.

**Table 4:** Logistic regression for HSES population.

Predictor	β	P-Value	Odds Ratio	95% Confidence Interval
Constant	3.316	<0.001		
Age	−0.133	<0.001	0.875	0.856–0.895
Age Squared	0.002	<0.001	1.002	1.002–1.003
BMI	−0.002	0.671	0.998	0.991–1.006
BMI Squared	<0.001	0.789	1.000	1.000–1.000
Weight (Wt) Gain	−0.047	<0.001	0.954	0.952–0.955
Weight Gain	<0.001	<0.001	1.000	1.000–1.000
Married	−0.172	<0.001	0.842	0.821–0.864
Previous Births	−0.217	<0.001	0.805	0.793–0.817
Prenatal Care Initiation (Reference = No Prenatal Care)				
*First Trimester*	−0.814	<0.001	0.443	0.409–0.479
*Second Trimester*	−0.931	<0.001	0.394	0.363–0.428
*Third Trimester*	−1.121	<0.001	0.326	0.296–0.358
HTN	1.129	<0.001	3.092	3.033–3.152
DM	0.261	<0.001	1.299	1.269–1.329
Race (Reference = White)				
*Black*	0.356	<0.001	1.428	1.389–1.468
*Hispanic*	0.149	<0.001	1.161	1.132–1.190
* Other Races*	0.103	<0.001	1.108	1.085–1.133

**Table 5:** Logistic regression for LSES population.

Predictor	β	P-Value	Odds Ratio	95% Confidence Interval
Constant	1.646	<0.001		
Age	0.046	0.005	1.048	1.014–1.082
Age Squared	<0.001	0.353	1.000	0.999–1.000
BMI	−0.081	<0.001	0.923	0.915–0.931
BMI Squared	0.001	<0.001	1.001	1.001–1.001
Weight (Wt) Gain	−0.026	<0.001	0.974	0.972–0.976
Weight Gain	<0.001	<0.001	1.000	1.000–1.000
Married	−0.135	<0.001	0.874	0.853–0.895
Previous Births	−0.014	0.449	0.986	0.951–1.022
Prenatal Care Initiation (Reference = No Prenatal Care)				
*First Trimester*	−0.864	<0.001	0.422	0.393–0.452
*Second Trimester*	−1.028	<0.001	0.358	0.333–0.384
*Third Trimester*	−1.228	<0.001	0.293	.270–0.318
HTN	1.058	<0.001	2.880	2.789–2.974
DM	0.421	<0.001	1.523	1.474–1.573
Race (Reference = White)				
*Black*	0.215	<0.001	1.240	1.203–1.279
*Hispanic*	−0.081	<0.001	0.922	0.897–0.948
*Other Races*	−0.141	<0.001	0.868	0.827–0.911

## Discussion

The results indicate a minor obesity paradox (for Obesity stage 1) in PTB for mothers who are LSES. An obesity paradox is a phenomenon where higher BMIs are shown to be protective and associated with better outcomes than being normal weight. This has been demonstrated in other areas of medicine and epidemiology. In a study by Hainer, there was evidence of an obesity paradox with peripheral arterial disease where the overall mortality rates decreased with increasing BMI. Those who were of normal weight had a mortality of 50%. This was greater than the rates for those who were overweight and obese who had rates of 40% and 31% respectively [28]. Flegal et al found that for all-cause mortality, Grade one obesity overall was not associated with higher mortality, and overweight was associated with significantly lower all-cause mortality than being normal weight. This study did find that obesity grade two and three were associated with higher all-cause mortality demonstrating a similar curvilinear pattern that was seen in this study [29].

Similar results to this study were found in Sharifzadeh et al. The authors found that PTB had a negative correlation with maternal BMI (r = -0.124, p = 0.004). This study also found those who were underweight had a PTB rate of 42.9% while those who were overweight and obese had rates of 5.1% and 5% respectively [13]. In Kosa et al, the only BMI category that had significantly increased odds of PTB was the underweight group who had 2.11 greater odds of PTB than the normal weight group [11].

In general, the literature concerning maternal weight and PTB, has not demonstrated a consensus. Several studies that had findings contrary to the above results. According to the meta-analysis completed by McDonald et al, the risk of preterm birth appeared significantly higher in overweight and obese women (1.24, 1.13 to 1.37). This study included induced preterm birth, which may have been permitted as a matter of medical judgement as opposed to the premature rupture of the birth membranes [6]. Another study that had similar results as MacDonald was Khatibi et al who found that increased body mass index was associated with an increased risk of preterm delivery with adjusted odds ratios (aOR) ranging from 1.11 (95% confidence interval [CI], 1.03–1.20) for pre-obesity to 2.00 (95% CI, 1.48–2.71) for grade-III [9]. One thing that can account for the disconnect is that many studies examine BMI as a linear phenomenon. In our study, we found that BMI (as well as age and weight gain) does not have a linear relationship with PTB, but has a curvilinear association where there is an optimal point where the odds of PTB is at its lowest rate. In our study, we found that for LSES women, when BMI increases up to 26–32, odds of PTB decreases. Once one has a BMI that is greater than 32.5, it is no longer protective against PTB causing its odds to increase.

Another finding was that BMI was a significant factor for LSES women and not for HSES women. While the difference that was found has not been explored for PTB, there have been studies that examined weight differences between the SES’s. Paeratakul et al examined the perception of weight based on SES and found that those with a higher income (greater than 350% times the poverty rate) had a higher percentage perception of being overweight than those who were of a lower income (50.2% vs 44.2%) p = 0.002. They also found that those with higher education (HS graduate and above) had 1.6 greater odds of having a perception of being overweight than those with a lower education [26]. Given the results of this study, it could be concluded that those who have a higher perception of being overweight may work harder to lose weight. This finding is consistent with a meta-analysis by Sobal et al, who found that women of a higher SES diet more often than women of lower SES. This analysis also found that HSES women tend to be more committed to the view that slimness is desirable and may be more motivated to attain slim figures [27].

Given the differences in perceptions of weight, the reason why BMI may not be significant in HSES women is because they are all working toward an ideal of slimness regardless of weight. This is further facilitated by their access to resources that facilitate dieting, programs for weight control, more expensive foods that are perceived as aids to dieting and more leisure time that allows greater opportunity for recreational exercise. Whereas LSES women who are of lower BMI may be food insecure while those who are obese have greater access to food, giving them the nutrition needed to optimize their pregnancy outcomes.

### Clinical implications

While it is understood that extra monitoring should be done with pregnant women with higher BMI’s, there should be extra attention paid to those who are under and normal weight particularly if they are LSES. Given that nutritional status before pregnancy can have an influence on pregnancy outcomes, counseling and planning should be provided. During the planning, nutrition and lifestyle should be discussed. According to the ACOG, those who are pregnant need folic acid, iron, calcium and various vitamins. These can be ingested through the use of a pregnancy supplement. Addressing nutritional needs for LSES women can be done with the clinician in conjunction with a social worker to address potential food security and other social determinants of health. During the early stages of pregnancy, relevant blood tests may identify vitamin or other dietary deficiencies that may need to be supplemented and followed through the course of the pregnancy. Also, it is important that clinicians address implicit bias. In general, people who are of normal weight are often automatically regarded as healthier and not high risk for poor outcomes. Regardless, the above recommendations could potentially help reduce the rate of PTB within this sector of the pregnant population.

It should be noted that although study results showed evidence that obesity is protective against PTB in LSES women, that does not mean that there should be less care given to those who are obese. It just means that equal attention should be paid to both groups, particularly if they are LSES.

### Strengths and limitations of the study

The strength of our study was the wealth of data that we were able to obtain. We were able to get a full population of the births in the United States from the last 3 years and obtain over 10 million records. This data was very reliable as it was from the National Vital Statistics System of the National Center for Health Statistics. This organization received birth data from all 50 states and Washington, DC.

Some limitations of the study were that there was no specific PTB variable. It had to be derived from the data on gestational age. There was also a lack of data for income and occupation which led us to use education and insurance status to determine our SES. While this was an indirect way to determine the SES, these elements have been used in other studies as a measure.

## Conclusions

There were two aims of this paper. The first objective was to contribute to the literature by evaluating the relationship between BMI and PTB, as well as the relationship between BMI, PTB, and SES. The second objective was to show that BMI has a curvilinear relationship demonstrating an optimal weight where the odds of PTB are at its lowest. Our study found a minor obesity paradox in PTB for those women who are LSES at obesity level 1. This has the potential to change the practice of prenatal care of normal weight women. It was also found that the relationship between BMI and PTB is not linear but curvilinear indicating that after a certain BMI is reached, the relationship between these two elements changes from being inverse (as BMI increases, the odds of PTB decreases) to being a direct relationship (as BMI increases, the odds of PTB start to increase). We also found that while weight is a significant factor in LSES women, it is not significant in HSES women. This can be due to the SES differences in access to foods and lifestyles that lead to healthier weights.

## Supporting information

S1 File BMISES_Data_Part1. (7Z)

S2 File BMISES_Data_Part2. (7Z)
